# The Influencing Factors of Serum Lipids among Middle-aged Women in Northeast China

**Published:** 2018-11

**Authors:** Xinyao ZHANG, Li SHEN, Yanjun WANG, Xin GUO, Jing DOU, Yaogai LV, Zhiqiang XUE, Anning ZHANG, Lina JIN, Yan YAO

**Affiliations:** 1. Dept. of Social Medicine and Health Management, School of Public Health, Jilin University, Changchun, Jilin, China; 2. Dept. of Epidemiology and Biostatistics, School of Public Health, Jilin University, Changchun, Jilin, China; 3. Dept. of Nursing, Second Hospital of Jilin University, Changchun, Jilin, China

**Keywords:** Dyslipidemia, Influencing factors, Serum lipids, Quantile regression

## Abstract

**Background:**

Dyslipidemia is a common and serious health problem, especially in middle-aged women. We aimed to reveal quantile-specific associations of serum lipids [triglycerides (TG), total cholesterol (TC), low density lipoprotein cholesterol (LDL-c) and high density lipoprotein cholesterol (HDL-c)] with influencing factors in middle-aged women.

**Methods:**

A sample of 5635 participants were enrolled from Jilin, China, in 2012. Quantile regression (QR) model was performed to identify factors which influenced serum lipids in different quantiles.

**Results:**

The influencing factors of TG, TC, LDL-c and HDL-c were different. Waist circumference (WC), menopause, smoking, diabetes and hypertension were positively associated with TG in almost all quantiles; Menopause and age were positively associated with TC in almost all quantiles. WC, living in urban areas and alcohol consumption were positively associated with TC in low and middle quantiles, diabetes was positively associated with TC from P50 to P95. The result of LDL-c was similar to TC; BMI was negatively associated with HDL-c from P50 to P90. WC and diabetes were negatively associated with HDL-c from P5 to P90.

**Conclusion:**

Among middle-aged women, menopause, diabetes and WC were the main factors affecting the serum lipids. Postmenopausal women would get more risk in increasing the level of serum lipids.

## Introduction

A dyslipidemic profile, characterized by the elevated level of total cholesterol (TC), low density lipoprotein cholesterol (LDL-c) and/or triglycerides (TG), or low level of high density lipoprotein cholesterol (HDL-c) alone (1). In China, dyslipidemia is a common and serious health issue, with the prevalence increasing from 18.6% in 2002 to 40.4% in 2012 among adults (2, 3).

The occurrence of dyslipidemia was related to obesity, unhealthy lifestyles, age, decreased lipid metabolism, and lack of estrogen or glucocorticoid stimuli (4, 5). As dyslipidemia develops, intravascular lipid, cholesterol, and other substances are gradually deposited on the inner wall of vessels to form lipid plaques or fibrous plaques, and those will lead to narrow arterial lumen and wall hardening. If the pathological processes continue, a series of cardiovascular complications, such as myocardial infarction, arrhythmia, cerebral hemorrhage, cerebral infarction, hypertension, diabetes and retinopathy may occur (6–10), affecting quality of life, or even lead to death. Middle-aged women as a special group of people experiencing estrogen levels decline, many of them are obese, have unhealthy living habits and are susceptible to disease. Thus, it is of great importance to find the mechanism of dyslipidemia and its related influence factors in middle-aged women, to prevent and intervene serious diseases occurring caused by dyslipidemia.

At present, most researches on dyslipidemia were performed among adults (both male and female), with little attention to middle-aged women. Therefore, middle-aged women were used as the object of our study to explore serum lipids related factors.

Generally speaking, the occurrence and development of dyslipidemia is a continuous and long-term process (5, 11). In this case, factors may play different role in the process of dyslipidemia development. In practice, however, many studies analyzed dyslipidemia as a categorical variable by ordinary least squares regression model, which could only estimate the average levels of changes. Thus, appropriate methods should be got to approach the real value and satisfy the demand of design. Quantile regression (QR) model, has high flexibility for data modeling of heterogeneous condition distribution (12) and can also provide whole pictures of covariates effects by modeling a set of percentiles (13). Therefore, QR model is more suitable for exploring influence factors of serum lipids in the process of dyslipidemia development.

In our study, QR models were performed to explore independent factors associated with four indices of serum lipids such as TG, TC, LDL-c and HDL-c, respectively, when treated them as continuous variables among middle-aged women.

## Methods

### Participants

Data was derived from a cross-sectional study of adult chronic disease and its risk factors in a study conducted in Jilin, China, in 2012. A multistage, stratified, random cluster sampling method was used to select 23,050 subjects aged 18 to 79 yr old and lived in Jilin Province for more than 6 months (14). Overall, 5,635 middle-aged women (aged 40∼65 years old) (15, 16), with full information of four serum lipids indices (TG, TC, LDL-c and HDL-c) and no control over serum lipids were enrolled.

### Data Collection and Measurement

All the information was collected by trained investigators. These data included demographics (gender, age, etc.), health-related behaviors (smoking, drinking, etc.) and anthropometric measurements (height, weight, etc.). Serum lipids levels (TG, TC, LDL-c and HDL-c) were measured by MODULE P800 biochemical analysis machine (Roche Co., Ltd., Shanghai, China) in the morning after participants fasted for 10 or more hours overnight. Fasting blood glucose levels were measured by the Bayer Bai Ankang fingertip blood glucose monitor machine (Bayer, Leverkusen, Germany). The participants’ height, weight and waist circumference (WC) were measured though standardized protocol and process, with clothing but no shoes. Body mass index (BMI) was calculated by the following formula.

$$\text{BMI}=\text{Weight}\;(\text{kg})/\text{Height}\;({\text{m}}^{2})$$

### Assessment Criteria

According to the serum lipids status, high TG: TG≥1.7 mmol/L, high TC: TC≥5.2 mmol/L, low HDL-c: HDL-c<1.0mmol/L, high LDL-c: LDL-c≥3.4 mmol/L(17). Diabetes was defined as fasting blood glucose≥7.0 mmol/L or use the hypoglycemic agents or a self-reported history of diabetes (18). Hypertension was defined as resting systolic blood pressure (SBP)≥140 mmHg and/or diastolic blood pressure(DBP)≥90 mmHg and/or the use of hypotensor in the past two weeks (17). Current smoker was defined as a person who smoked more than one cigarette per day in the past 30 days (17). Drinker was defined as a person who consumed an average of more than one alcoholic drink per week in the past 30 days, including any form of alcohol (19).

### Ethics and consent

The study was approved by the Ethics Committee of the School of Public Health Jilin University, and prior to the investigation, it was necessary for each participant to have informed consent. All methods were performed in accordance with the relevant guidelines and regulations.

### Statistical Analysis.

The means±standard deviations (SD) and median (inter-quartile range, IQR) were utilized to describe the continuous variables, QR model was used to identify factors that influence the level of serum lipids in different quantiles. Statistical analysis was performed using the R version 3.3.3 (TUNA Team, Tsinghua University China). Statistical significance was set at *P*-value < 0.05.

## Results

Overall, 5,635 participants were enrolled, including 2,492 premenopausal women and 3,143 postmenopausal women. The median age of participants was 50.0 (IQR: 12.0) yr old. A total of 502 participants suffered with diabetes and 2,083 with hypertension. There were 480 drinkers, 803 smokers and 3,041 urban residents. The median value of WC was 81.0 (IQR: 13.2) cm and BMI was 24.3 (IQR: 4.6) kg/m^2^.

Table 1 shows the median and boundary values of four indices of serum lipids. The critical values of TG, TC, LDL-c, and HDL-c in the QR model were P59.2, P61.5, P69.4 and P11.4, respectively. Table 2 to 5 shows coefficients and 95% confidence intervals of factors of TG, TC, LDL-c and HDL-c in middle-aged women, respectively.

**Table 1:** Description and boundary values of serum lipids

***Serum lipids***	***Median (IQR)***	***Demarcation value (mmol/L)***	***Percentile (%)***
TG	1.49(1.20)	1.7	59.2
TC	4.90(1.35)	5.2	61.5
LDL-c	2.97(1.15)	3.4	69.4
HDL-c	1.38(0.48)	1.0	11.4

TG: triglyceride; TC: total cholesterol; LDL-c: low-density lipoprotein cholesterol; HDL-c: high-density lipoprotein cholesterol

**Table 2:** Quantile regression coefficients and 95% confidence intervals for TG

***Factors***	***P5***	***P 10***	***P 25***	***P 50***	***P 59.2***	***P 75***	***P 90***	***P 95***
Menopause	0.127^*^	0.153^*^	0.187^*^	0.300^*^	0.329^*^	0.345^*^	0.347^*^	0.248
	(0.098,0.182)	(0.093,0.188)	(0.129,0.243)	(0.231,0.364)	(0.254,0.429)	(0.233,0.444)	(0.141,0.596)	(−0.237,0.992)
WC	0.010	0.011^*^	0.017^*^	0.027^*^	0.031^*^	0.038^*^	0.042^*^	0.075^*^
	(−0.337,0.002)	(0.008,0.014)	(0.013,0.020)	(0.022,0.030)	(0.027,0.035)	(0.029,0.045)	(0.032,0.060)	(0.014,0.098)
Hypertension	0.023	0.043	0.090^*^	0.083^*^	0.137^*^	0.242^*^	0.494^*^	0.477^*^
	(−0.023,0.078)	(−0.007,0.113)	(0.024,0.153)	(0.008,0.185)	(0.038,0.235)	(0.114,0.366)	(0.208,0.765)	(0.031,1.210)
Diabetes	0.153^*^	0.169^*^	0.270^*^	0.552^*^	0.648^*^	0.771^*^	1.713^*^	2.246^*^
	(0.042,0.224)	(0.102,0.262)	(0.147,0.393)	(0.296,0.762)	(0.491,0.767)	(0.588,1.061)	(0.844,2.643)	(1.227,4.494)
Smoke	0.132^*^	0.072^*^	0.225^*^	0.300^*^	0.314^*^	0.418^*^	0.663^*^	1.891^*^
	(0.068,0.165)	(0.029,0.182)	(0.126,0.309)	(0.169,0.447)	(0.190,0.432)	(0.168,0.558)	(0.191,2.259)	(0.890,4.150)

* P<0.05; WC:Waist circumference; BMI:Body mass index

**Table 3:** Quantile regression coefficients and 95% confidence intervals for TC

***Factors***	***P5***	***P 10***	***P 25***	***P 50***	***P 61.5***	***P 75***	***P 90***	***P 95***
Menopause	0.236^*^	0.297^*^	0.433^*^	0.364^*^	0.395^*^	0.383^*^	0.426^*^	0.536^*^
	(0.055,0.384)	(0.116,0.399)	(0.255,0.556)	(0.251,0.514)	(0.284,0.536)	(0.265,0.558)	(0.132,0.837)	(0.344,0.959)
WC	0.002	0.005^*^	0.010^*^	0.009^*^	0.009^*^	0.005^*^	0.001	0.004
	(−0.001,0.010)	(0.001,0.014)	(0.002,0.014)	(0.004,0.013)	(0.005,0.014)	(0.000,0.012)	(−0.004,0.017)	(−0.008,0.016)
Diabetes	0.006	0.032	0.062	0.213^*^	0.280^*^	0.304^*^	0.380^*^	0.530^*^
	(−0.199,0.134)	(−0.285,0.182)	(−0.095,0.252)	(0.044,0.364)	(0.106,0.474)	(0.167,0.520)	(0.136,0.827)	(0.247,1.049)
Alcohol consumption	0.358^*^	0.250^*^	0.219^*^	0.159^*^	0.178^*^	0.149	0.346	0.486^*^
	(0.113,0.482)	(0.112,0.371)	(0.073,0.308)	(0.066,0.262)	(0.032,0.302)	(−0.029,0.402)	(−0.011,0.893)	(0.270,1.374)
Age	0.017^*^	0.013^*^	0.010^*^	0.017^*^	0.022^*^	0.027^*^	0.030^*^	0.025
	(0.007,0.027)	(0.007,0.023)	(0.001,0.022)	(0.008,0.026)	(0.009,0.032)	(0.012,0.036)	(0.005,0.048)	(−0.001,0.048)
Urban	0.108^*^	0.199^*^	0.170^*^	0.184^*^	0.210^*^	0.146^*^	0.068	0.079
	(0.007,0.223)	(0.039,0.276)	(0.043,0.255)	(0.098,0.275)	(0.102,0.304)	(0.050,0.247)	(−0.110,0.259)	(−0.213,0.305)

* P<0.05; WC:Waist circumference

**Table 4:** Quantile regression coefficients and 95% confidence intervals for LDL-c

***Factors***	***P_5_***	***P _10_***	***P _25_***	***P _50_***	***P _69.4_***	***P _75_***	***P _90_***	***P _95_***
Menopause	0.248^*^	0.260^*^	0.266^*^	0.367^*^	0.359^*^	0.390^*^	0.488^*^	0.593^*^
	(0.081,0.322)	(0.113,0.360)	(0.137,0.377)	(0.213,0.484)	(0.243,0.470)	(0.252,0.538)	(0.331,0.681)	(0.341,0.781)
WC	0.003	0.006^*^	0.008^*^	0.010^*^	0.012^*^	0.013^*^	0.013^*^	0.013
	(−0.004,0.008)	(0.003,0.011)	(0.005,0.013)	(0.005,0.014)	(0.008,0.016)	(0.008,0.018)	(0.008,0.023)	(−0.000,0.025)
Diabetes	−0.218	−0.096	−0.113	0.092	0.203^*^	0.224^*^	0.286^*^	0.162^*^
	(−0.363,−0.017)	(−0.322,0.078)	(−0.204,0.016)	(−0.071,0.242)	(0.078,0.358)	(0.102,0.388)	(0.140,0.491)	(0.046,0.296)
Age	0.006^*^	0.009^*^	0.013^*^	0.010^*^	0.015^*^	0.011^*^	0.012	0.012
	(0.002,0.020)	(0.001,0.019)	(0.005,0.020)	(0.003,0.021)	(0.007,0.021)	(0.002,0.023)	(−0.005,0.023)	(−0.006,0.034)
Urban	0.045	0.096^*^	0.182^*^	0.238^*^	0.256^*^	0.244^*^	0.209^*^	0.009
	(−0.023,0.217)	(0.037,0.194)	(0.103,0.243)	(0.182,0.334)	(0.164,0.335)	(0.161,0.351)	(0.107,0.357)	(−0.111,0.382)

* *P*<0.05; WC:Waist circumference

**Table 5:** Quantile regression coefficients and 95% confidence intervals for HDL-c

***Factors***	***P_5_***	***P _10_***	***P _11.4_***	***P _25_***	***P _50_***	***P _75_***	***P _90_***	***P _95_***
BMI	0.001	0.001	−0.003	−0.001	−0.004^*^	−0.013^*^	−0.014^*^	−0.033^*^
	(−0.011,0.009)	(−0.009,0.004)	(−0.007,0.005)	(−0.008,0.004)	(−0.011,−0.003)	(−0.023,−0.004)	(−0.035,−0.000)	(−0.044,−0.019)
WC	−0.007^*^	−0.007^*^	−0.006^*^	−0.007^*^	−0.008^*^	−0.007^*^	−0.007^*^	−0.003
	(−0.011,−0.002)	(−0.009,−0.004)	(−0.010,−0.004)	(−0.010,−0.005)	(−0.011,−0.006)	(−0.010,−0.004)	(−0.012,−0.001)	(−0.009,0.001)
Diabetes	−0.057^*^	−0.069^*^	−0.072^*^	−0.110^*^	−0.096^*^	−0.145^*^	−0.087^*^	−0.119
	(−0.146,−0.013)	(−0.112,0.034)	(−0.115,−0.047)	(−0.153,−0.065)	(−0.134,−0.065)	(−0.186,−0.079)	(−0.173,0.004)	(−0.169,0.007)

* *P*<0.05; WC:Waist circumference; BMI:Body mass index

Menopause was positively associated with TG from P5 to P90. WC was positively associated with TG from P10 to P95. Hypertension was positively associated with TG from P25 to P95. Diabetes and smoking were positively associated with TG in all percentiles, moreover, coefficients were increasing as TG increasing. The coefficients of diabetes, hypertension and smoking increased rapidly from P59.2 to higher levels.

As shown in Table 3, menopause was positively associated with TC in all percentiles, and coefficients increased as TC increasing. WC was positively associated with TC from P10 to P75. Age was positively associated with TC from P5 to P90. Diabetes was positively associated with TC from P50 to P95, and coefficients increased as TC increasing. Compared with living in rural area, living in urban area was positively associated with TC from P5 to P75. Alcohol consumption was positively associated with TC as percentile less than 61.5.

Menopause was positively associated with LDL-c in all percentiles, and coefficients increased as LDL-c increasing (Table 4). WC and living in urban area were positively associated with LDL-c from P10 to P90. Age was positively associated with TC from P5 to P75. Diabetes was positively associated with LDL-c from P69.4 to higher levels and coefficients increased as LDL-c increasing.

As seen in Table 5, BMI was negatively associated with HDL-c from P50 to P95. WC and diabetes were negatively associated with HDL-c from P5 to P90.

## Discussion

We found that among middle-aged women, menopause, diabetes and WC were positively associated with TG, TC and LDL-c; BMI, WC and diabetes were negatively associated with HDL-c. Besides, hypertension was positively associated with TG. And on different levels of TG, TC, LDL-c and HDL-c, the extents of the effects of each factor were different.

In our study, menopause was positively associated with TG, TC and LDL-c, and the coefficient increased as percentile increasing, especially in high quantiles of TC and LDL-c. That means, as TC rising to higher levels, the associations between menopause and TC were getting more and more stronger, as was also the case with LDL-c. The reason for this was probably because of a decline in estrogen levels among postmenopausal women (20, 21). Estrogen plays an important role in serum lipids, it can reduce TG synthesis, increase liver uptake of LDL-c and secretion of cholic acid, accelerate cholesterol removal in vivo, thereby reducing the serum TG, TC and LDL-c levels (20, 22). In addition, when estrogen levels drop, many women will experience weight gain. Redistribution of adipose tissue leads to an increase in abdominal fat deposition (23). WC is an important indicator of central obesity, our study showed that WC was an independent risk factor of TG, LDL-c and HDL-c in almost all quantiles, and TC from P10 to P75. That means, as TG, LDL-c and HDL-c elevating to high levels, they will be more sensitive to the increasing of WC. Those important information cannot be found in traditional model when serum lipids were treated as a categorical variable.

A previous study, indicated a close relationship between diabetes and dyslipidemia (24). In our study, diabetes was an independent risk factor of increased TG and decreased HDL-c in almost all quantiles, with TC from P50 to P95, and with LDL-c from P69.4 to P95. Furthermore, the coefficients increased as TG, TC and LDL-c increasing. That means, as TG, TC, LDL-c and HDL-c elevating to high levels, all the four indices of serum lipids will be more sensitive to diabetes. These probably because patients with diabetes were more likely to had lipid metabolic disorders, which may be associated with insulin resistance and insulin sensitivity (25), while increasing serum lipids levels would lead to dysfunction of pancreatic β-cell function (26). This indicated that patients with diabetes should pay high attention to serum lipids.

In our study, we also found that hypertension was associated with TG in all quantiles, and as TG increasing, the association were getting stronger, especially in high quantiles of TG (P59.2 to P95). Hypertension was related with disorder of lipid metabolism (27), in turn, TG can be used as an important factor in predicting hypertension (28). Therefore, middle-aged women with hypertension would attain more risk in TG. Some limitations should be noted in present study. Firstly, this was a cross-sectional study and participants were selected from Jilin Province, hence, selection bias could exist and limit the results generalize to other populations. Secondly, some information was collected by self-report, such as smoking and alcohol consumption, thus, social desirability bias may be present and underestimate the associations between these factors and serum lipids. Thirdly, some potential confounders were not under our consideration, such as gene and physical activity, which might have some effects on our results.

## Conclusion

On different levels of TG, TC, LDL-c and HDL-c, the influence degree of each factor was different. Among middle-aged women, menopause, diabetes and WC were the main factors affecting the serum lipids. With the increasing of WC, they would get a higher level of serum lipids. And postmenopausal women would get more risk in increasing the level of serum lipids.

## Ethical considerations

Ethical issues (Including plagiarism, informed consent, misconduct, data fabrication and/or falsification, double publication and/or submission, redundancy, etc.) have been completely observed by the authors.
